# Frequency of transfusion transmissible infections among blood donors of Rawalpindi District, Pakistan

**DOI:** 10.4314/ahs.v22i3.63

**Published:** 2022-09

**Authors:** Saqlain Ghazanfar, Sarmad Hassan, Zia Shahid, Muhammad Sheharyar Khan, Abdur Rehman Malik, Hamza Sattar Bhutta, Nadeem Ikram, Muhammad Sarfraz Khan

**Affiliations:** 1 Final Year Medical Student, Rawalpindi Medical University; 2 Medical Graduate, Rawalpindi Medical University; 3 Fourth Year Student, Rawalpindi Medical University; 4 Assistant Professor of Pathology and Microbiology, Rawalpindi Medical University

**Keywords:** Transfusion, Hepatitis, HIV, Syphilis, Malaria, Pakistan

## Abstract

**Background:**

Transmissible Infections (TTI's) are a cause of significant burden on health care facilities by imposing a threat of infection transmission through disease reservoirs in asymptomatic donors. This eventually leads to a serious challenge in acquiring blood bags in a country like Pakistan where transfusion dependent disease are of high prevalence. The objective of this study is to determine the seroprevalence of TTI's in blood donors in Rawalpindi District through a multi-center approach.

**Materials and Methods:**

This is an observational descriptive retrospective study based on 6 transfusion centers in the Rawalpindi District. The time frame of the study was from January 2015 to December 2018. A total of 223,242 donors were consecutively included and data on donor type, the purpose of transfusion, and seroprevalence (HBV, HCV, HIV, Syphilis, and Malaria) were collected through a structured questionnaire and laboratory investigation results. The collected data were entered in SPSS version 21.0 for analysis.

**Results:**

The seroprevalence of blood borne infections was 7,897 (3.537%) of which HBV, HCV, HIV, Syphilis and Malaria accounted for 2410 (1.080%), 3105(1.391%), 0(0.000%), 2017 (0.933%) and 365 (0.171%), respectively. Reactive samples reduced from 4.850% to 3.537% over 4 years, while there was a rise of 37.478% of blood donors from 2015 to 2018. The total number of voluntary donors and replacement donors was 22079 (9.890%) and 201156 (90.107%), with a rising incidence in voluntary donors from 2015 to 2018. A considerable number of donor bags were transfused to Thalassemia, Anemia, Leukemia and Hemophilia patients, 28156 (12.612%). This number also showed increasing rates from 11.654% to 14.017%.

**Conclusion:**

In conclusion, our study suggests that the risk of transmission through transfusion is still considerable. Targeting donors with a low-risk profile, a screening questionnaire, an ample supply of quality screening tests, and awareness campaigns for the diseases in question must be carried to further decrease the risk of transmission of TTIs in Pakistan.

## Introduction

Blood transfusions are an essential part of patient care in trauma and critical settings^1^. According to WHO, “Blood is a universal right”. It can save the lives of millions of people worldwide from acute as well as chronic illnesses. Today, the use of whole blood is a well-accepted and commonly employed measure, without which many modern surgical procedures could not be carried out^2^. But all these advantages come with the potential of transmitting a collection of debilitating diseases through these blood products if improperly screened. The diseases that can be transmitted through blood include Hepatitis B, Hepatitis C, Acquired Immunodeficiency Syndrome (AIDS), Syphilis, Malaria, and many more^3^.

More than 17.4 million units of blood are donated every year in the world^4^. In Pakistan, more than 1.5 million pints of blood are collected each year. Among them, replacement donors, voluntary donors, and professional donors account for 65%, 25%, and 10%, respectively^5^. A study regarding Transfusion Transmissible Infections was done in Pakistan in 2012. This study reported prevalence for Hepatitis B surface antigen (HBsAg), anti-HCV, anti-HIV, and Syphilis to be 2.68%, 2.46%, 0.06%, and 0.43% respectively, with an increasing trend in frequencies of transfusion-transmitted infections (TTIs).

However the study was based on a single center in Peshawar^6^. Very limited studies have been conducted within Pakistan as well as internationally which would report TTI's. Not only does this pose a threat to the patients who receive transfusion on need basis but also puts a burden on the economy of countries which is afflicted due to raised cost of provision of health care to these patients. Joint United Nations Programme on HIV and AIDS (UNAIDS) has estimated that only 50 % of the 1.5 million blood bags are screened in Pakistan^7^. Keeping this data into consideration, it seems crucial to screen the blood for TTIs to minimize further the risk of transmitting potentially fatal disease through the blood.

There is a dire need to increase the number of safe blood products available for emergency use. Furthermore, this increase should complement the proper screening of the donated blood to minimize the risk of spreading transfusion-transmissible infections. The trends are an ever-changing thing, and so is the literacy rate and the mindset of people. The basic premise of our study was to provide an epidemiological analysis of Transfusion Transmissible Infections (TTIs) amongst the blood donors of Rawalpindi District, Pakistan. Our study also aimed to determine the prevalence of HIV, Hepatitis B, Hepatitis C, Malaria, and Syphilis amongst healthy blood donors of Rawalpindi. Adding further, percentage of transfusions aimed to provide blood bags to patients with blood-related disorders was also aimed to be calculated as a part of this study.

## Design and Methodology

### Study Design

It is a retrospective observational study based on the data obtained from the blood banks of Holy Family Hospital, Benazir Bhutto Hospital, District Headquarter Hospital Rawalpindi, Tehsil Headquarter Hospital Murree, Samli TB Sanitorium, and Jamila Sultana Foundation. The duration of this study was from 2015–2018. Data were obtained by permission of the blood bank in-charge of the respective blood banks.

### Study Population

The study included the screening results of 223,242 blood donors. They donated blood in any of the above-mentioned blood banks between 2015 and 2018. The donors included volunteer donors, who gave blood voluntarily to the blood banks, and replacement donors, who were called upon to donate blood to the patients. However, the data did not segregate the two categories. According to the blood donors' guidelines of WHO, males with Hb level less than 12.5 g/dl and females with Hb level less than 12 g/dl were not allowed to donate blood. Similarly, the donors who had a recent history of active TTI were not permitted to donate blood.

According to the blood donors' protocol of the government of Pakistan, all the blood donors were screened for TTI, including HBV, HCV, HIV, Syphilis, and Malaria.

### Laboratory Procedures

Blood Collection: Venous blood of the donors was obtained with a 5mm syringe in an EDTA-containing vile to be screened for anti-HIV (1&2), anti-Syphilis, Hepatitis B surface antigen (HBsAg), anti-Hep C, and Malaria PF/PAN antigen.

### Statistical Analysis

The collected data were entered and analysed in SPSS version 21.0. Frequencies and percentage, summary statistics were computed for all the variables and presented in the form of tables. The results were also represented in line diagrams to show trends in seroprevalence from 2015 to 2018. Chi-square/Fischer exact test was applied to check if any significant difference exist among prevalence of different pathogen over the years.

## Results

From January 2015 to December 2018, 223,242 blood donors were analyzed among the general population, among which 201,156 (90.10%) were replacement donors, while 22,079 (9.9%) were voluntary donors. There was a rise of 37.478% donors from year 2015 to 2018, and a rise in percentage of voluntary donors. Table 2 shows the year-wise percentages of replacement and voluntary donors.

**Table 2:** Distribution of Voluntary and Replacement Donors

Year	Total donors	VD*	%	RD**	%
2015	45854	4276	9.3252	41578	90.6748
2016	55558	5069	9.1238	50482	90.8636
2017	58791	5750	9.7804	53041	90.2196
2018	63039	6984	11.0789	56055	88.9211
P-value	0.176***	0.231***		0.099***	
Total	223242	22079	9.89016	201156	90.1067

* Voluntary Donors

** Replacement Donors

*** Fischer Exact Test (Year vs total donor/donor type)

All the donors were screened for Hep B, Hep C, and HIV during these four years. Total number of samples that came out positive for Hep B, Hep C & HIV were 2410 (1.07%), 3105 (1.39%) and 0 (0%) respectively. The total number of samples screened for Syphilis, and Malarial parasite was 216190 & 213902, respectively. The total number of samples that came out positive was 2017 (0.93%) and 365 (0.17%). From 2015 to 2018, none of the donors tested positive from HIV. The year-wise prevalence of Hep B, Hep C, Syphilis and Malaria is given in Table 3

**Table 3:** Screening Results

			Hepatitis B		Hepatitis C		Syphilis		Malaria	
Year	Total donors	Total Reactive (Percentage)	Total screened	HBV +ve, (Percentage)	Total screened	HCV +ve (Percentage)	Total screened	Syphilis +ve, (Percentage)	Total screened	MP +ve (Percentage)
2015	45854	2224, (4.85%)	45854	602, (1.31%)	45854	935 (2.04%)	39176	542, (1.38%)	38534	145, (0.38%)
2016	55558	2058, (3.70%)	55558	621, (1.12%)	55558	810 (1.46%)	55355	524, (0.95%)	53819	103, (0.19%)
2017	58791	1856, (3.16%)	58791	610, (1.04%)	58791	683 (1.16%)	58791	511, (0.87%)	58791	52, (0.09%)
2018	63039	1759, (2.79%)	63039	577, (0.92%)	63039	677 (1.07%)	62868	440, (0.70%)	62758	65, (0.10%)
P-value	0.472*	0.341*	-	0.134*	-	0.176*	-	0.289*	-	0.341*
	223242	7897 (3.54%)	223242	2410, (1.08%)	223242	3105 (1.39%)	216190	2017, (0.93%	213902	365, (0.17%)

* Fischer Exact Test

**Figure 1:**
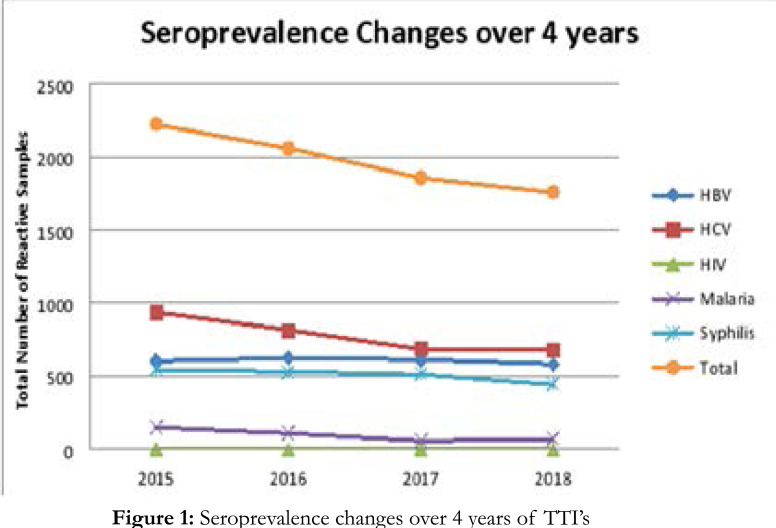
Seroprevalence changes over 4 years of TTI's

The total number of positive samples for one or multiple TTIs during these four years was 7897 (3.537%) as summarized in Table 3. The seroprevalence reduced over the years for all the infections from 2224 reactive samples in 2015 to 1759 reactive samples in 2018, as shown by figure 1. This decrease in reactive samples was non-significant as shown in table 3. Among the individual diseases, trend analysis did not given any significant differences.

Blood demand for certain blood-related disorders (Thalassemia, Hemophilia, Leukemia & anemia) is increasing day by day as the number of blood donations for these diseases increases every year (P-value=0.098) (Table 4).

**Table 4:** Demand for Blood Donation for Blood-Related Disorders

Year	Total donors	Transfusions going to thalassemia, Leukemia, anemia, hemophilia pts	%
2015	45854	5344	11.65
2016	55558	6201	11.16
2017	58791	7775	13.22
2018	63039	8836	14.02
	223242	28156	12.61
**P-value**	0.098*		

* Fischer exact test

## Discussion

Pakistan is endemic for many TTI's while the demand for blood donation is increasing on an annual basis, which is evident from our results. Thus, the frequent transfusion of blood to these patients increases the chances of being affected by a TTI considerably. Therefore, strict screening procedures should be carried out all over the country before the blood is transfused.

In our study, 90.10% of donors were replacement donors, while 9.9% were voluntary donors. Similar studies in Pakistan have come up with comparable figures, such as the study conducted by Arshad and Saeed et al. in Karachi and Lahore, respectively^8^,^9^. In contrast, studies in other Asian countries show a more significant number of voluntary donors. A study conducted in India showed that 65.3% were voluntary donors, and 34.7% were replacement donors. The study also showed a sharp rise in the yearly rates of voluntary donors rising from 15.2% to 94 % over ten years (i.e., 2004–13). In contrast, the number of voluntary donors in our study remained relatively consistent. Sharma et.al attributed this sharp rise in voluntary donors to the effort of government institutions to raise public awareness^10^. A study by Stockx et al. in Mozambique also found a high percentage (50.5%) of voluntary donors^11^. However, a survey by Chaudhary et al. conducted in Uttar Pradesh India found only 39.47% of their donors to be voluntary. Although lower than Sharma's study, these numbers are still comparatively higher than those of Pakistan. The low prevalence of voluntary blood donors in Pakistan represents a general lack of awareness and health education. Furthermore, there could be fears and misconceptions in the public related to donating blood.

Our study focused on four main TTIs. The overall seroprevalence of HBV was 1.93 %. Similar results have been reported in studies conducted all over the country such as by Memon et al. (1.4%) in Hyderabad (2017)^12^, Awan et al. (1.09%) in Islamabad (2018) (13), Arshad et al. (1.7%) in Karachi (2016)^8^ and Saeed et al. (1.1%) in Lahore^9^. Hussain et al., however, reported a slightly higher figure of 2.32% in Multan (2015)^14^. Internationally, a study in Uttar Pradesh reported a comparable number of 1.93% (2014)^15^. Several studies showed a comparatively lower prevalence for HBV, such as the one done by Farshadpur et al. in Iran (2016)^16^. The survey conducted by Zou et al. in the U.S.A in 2009 showed an even lower number (1 for every 280,000)^17^. The reason for that is probably the high level of awareness among the population and generally low prevalence. A study in Namibia in 2014 also found a low figure of 0.6%, which could be due to the small sample size of the study^18^. This is because most of the results reported from all over Africa are considerably higher, ranging from 5%–14.96% in various countries of the continent^11^,^19^–^22^. In a study conducted in 2011, Nagalo et al. attributed their particularly high figure (14.96%) to the fact that the majority of their donors belonged to the rural population as well as the marked use of tattooing among the Mossi, Gourounsi, and Bobo, the major ethnic groups in the Koudougou region^19^. Tattooing has been further confirmed as a significant risk factor for HBV transmission by Jombo et al.^20^.

In this study, we found the prevalence of HCV to be 1.39%, which is similar to that of various local studies i.e., 1.3% (13) and 1.8%^8^. On the other hand, the other local studies all reported higher numbers of 3.52%^11^, 2.62%^9^, and 3.44%^14^. This is alarming as HCV is extremely dangerous, and the true extent of its prevalence in our society is still yet to be determined. Looking outwards, a study in Ethiopia and one in India found somewhat similar figures of 1.7% and 1.02, respectively^14^,^22^, while Nagalo et al. reported a towering figure of 8.69%^19^. On the other hand, most of the studies showed a reported lower value i.e., 0.1% in Iran and Namibia, 0.7% in Eritrea, and 0.36% in Gwalior^10^,^16^,^18^,^22^.

In this study, we did not find a single HIV+ patient during our 4-year study period. This was, regrettably, not the case for many other studies, as most of them reported HIV+ percentages ranging from 0.01–0.16%^8^,^9^,^12^–^14^. Waheed et al., however, conducted a study in 2012, where they reported an HIV prevalence of 0% in Islamabad^24^. This could be attributable to screening variations in different locations and the fact that screening tests lack complete sensitivity. Farshaadpour et al. reported a similarly low figure of 0.004% from Iran^16^. Others have reported higher numbers ranging from 0.13% in Gwalior, India, to 0.8 % in Eritrea^10^,^15^,^18^,^21^,^22^. Studies from other parts of Africa have reported even higher numbers, namely Burkina Faso (2.21%) and Mozambique (8.5%)^11^,^19^. This is not surprising, as it coincides with the fact that the general prevalence rates for Africa are highest in the world^25^.

Moving on, the prevalence rate for Syphillis was 0.93%. Awan and Waheed et al. found a comparable rate of 0.75% and 0.89% in separate studies in Islamabad^13^,^24^. Figures for Lahore, Karachi, and Hyderabad were all higher, i.e., 1.55%, 2.1%, and 3.01%, respectively^8^,^9^,^12^. This could reflect a difference in the level of awareness about the disease in a particular region. Hussain et al., however, reported a considerably low figure of 0.07%. Internationally, Stokx et al. reported a similar figure of 1.2%. Mavenyengwa et al. reported a lower figure of 0.3%, while India reported even lower prevalence rates of 0.16% and 0.17% form Uttar Pradesh and Gwalior, respectively^10^,^15^,^18^. On the other hand, figures from different parts of Africa were considerably higher, i.e., 3.96% in Burkina Faso and 7.2% in Eritrea^19^,^22^. The study by Nagalo et al. was primarily conducted in the rural setting, which could account for the higher prevalence rates. At the same time, Keleta et al. postulate their result could be suggestive of a high-density carrier state or active infection^19^,^22^.

Finally, our results showed a prevalence rate of 0.17% for Malaria. Most developed countries around the world do not screen for Malaria. However, in countries where the disease is endemic, it is still a notable transfusion transmissible reaction. Thus, strict screening is carried out for Malaria, as it poses a significant threat to the local population. Ali et al. reinforce this point by saying that blood donors in developing countries mostly comprise a low-income group and are commercial donors, which significantly increase transmission risk^26^. They also reported a considerably higher prevalence rate of Malaria (0.577%) in Peshawar's blood donors^26^.

On the other hand, Hussain, Awan, and Arshad et al. reported considerably lower figures of 0.07% (14), 0.02%^13^ and 0.07%^8^ respectively but data reported by Saeed et al. (0.1%) and Memon et al. (0.1%) were more comparable to our study^9^,^12^. Hussain et al. attribute this difference of results to the primary geographical and climatic variations that directly affect the reproduction of the parasite^14^. A study in Nigeria corroborated this fact as they found a considerable variation in their prevalence rates depending upon the season. It varied from an average of 26.92% in the dry season to an average of 55% during the rainy season^27^. The extremely high prevalence in Nigeria is also reported in a study by Achidi et al.^28^. According to Okocha et al., such a high prevalence rate reflects the high rate of asymptomatic malaria parasitemia in endemic malaria regions, which reinforces the need for strict screening practices in these regions^27^. Gelaw et al. reported a much closer number of 1% from Ethiopia, although it is still considerably higher than our results^23^. In contrast to this, studies from India and Sudan reported significantly lower prevalence rates of 0.03% and 0.056%, respectively^10^,^29^.

Although our study was conducted over a substantial period, it was a retrospective study. Thus, all of the limitations inherent to retrospective studies apply here as well. Furthermore, our focus was mainly the urban setting. The prevalence of the surrounding rural areas might well be comparatively different, and studies focusing on those areas are needed. Furthermore, only rapid screening tests were used, and no confirmatory testing by PCR was performed. The actual prevalence rates may be somewhat different from those found here. However, the current results are adequate to give us an approximate idea of the prevalence of TTI's in the study population.

## Conclusion

our study suggests that the risk of transmission through transfusion is still considerable. However, the decreasing trends seen throughout the study are heartening as they show a growing awareness about the diseases. They also show the effectiveness and the necessity of the continuation of strict screening protocols, which is supported by other studies as well. Thus, the current efforts must be continued, while further awareness about the benefits of blood donation must be raised to increase the population of voluntary donors. Furthermore, targeting donors with a low-risk profile, a screening questionnaire, an ample supply of quality screening tests, and awareness campaigns for the diseases in question must also be carried to further decrease the risk of transmission of TTIs in Pakistan.

## Figures and Tables

**Table 1:** Blood testing Kits Used

Screening	Device & Method	Detection in Serum/Plasma	Sensitivity	Specificity
**HIV**	Advanced Quality TM one step Test, Rapid Anti-HIV (1&2); Immunochromatography method	Antibodies against HIV (1&2)	99.80%	100.00%
**Syphilis**	ImuMed TM, One Step test; Immunochromatography method	Anti-Treponema pallidum antibodies	89.20%	90.90%
**HBV**	Quindao Hightop Biotech Co., Ltd. HBsAg Rapid Test Cassette (S/P); Immunochromatography method	Hepatitis B surface antigen	95.90%	96.40%
**HCV**	Healgen Scientific LLC, HCV Rapid Test Cassette; Immunochromatography method	Anti- HCV antibodies	98.10%	98.90%
**Malaria**	Accurate TM Malaria PF/PAN (ICT) Test Device (Whole Blood); Immunochromatography method	Plasmodium falciparum and Plasmodium Vivax antigen	92.50%	98.30%
